# Evaluating the effects of antimicrobial stewardship program on antimicrobial consumption and resistance patterns: a quasi-experimental study

**DOI:** 10.1186/s12879-026-13358-8

**Published:** 2026-05-20

**Authors:** Noha Morsi, Eleia Mosaad, Omar Ahmed

**Affiliations:** 1 Clinical Pharmacy Department, Aswan Heart Centre, Magdi Yacoub Foundation, Kasr El Hagar Street, Aswan, 81511 Egypt; 2 Adult Critical Care Department, Aswan Heart Centre, Magdi Yacoub Foundation, Aswan, Egypt

**Keywords:** Antimicrobial stewardship, Antimicrobial resistance, Antibiotic consumption, Defined daily dose, Multidrug-resistant organisms

## Abstract

**Background:**

Antimicrobial stewardship programs (ASPs) are coordinated efforts designed to promote the judicious use of antimicrobials, decrease microbial resistance, reduce the spread of multidrug-resistant infections, and improve patient outcomes. This study aimed to evaluate the impact of an ASP on antimicrobial consumption and antimicrobial resistance in a tertiary cardiac centre in Egypt.

**Methods:**

A quasi-experimental study was conducted in the adult critical care unit of Aswan Heart Centre, Magdi Yacoub Foundation, Aswan, Egypt to assess the changes in antimicrobial consumption and resistance patterns before and after ASP implementation. All patients admitted to the adult critical care area from July 2023 to 30 June 2024 were included in the study.

**Results:**

A total of 1000 patients were included in the pre-intervention period and 982 in the post-intervention period. Following ASP implementation, overall antimicrobial consumption decreased by 23% (from 14,414 to 11,012 DDDs per 1,000 patient days), although this was not statistically significant (*p* = 0.097). Colistin use declined significantly (817 to 53 DDDs per 1,000 patient days; *p* = 0.006). Infections caused by *Klebsiella pneumoniae* decreased significantly (42 to 19 isolates; *p* = 0.004). Among Enterobacteriaceae, *Escherichia coli* showed improved susceptibility to gentamicin (36% to 86%; *p* = 0.01) and meropenem (64% to 100%; *p* = 0.02). Reductions in fungal infections and improvements in antifungal susceptibility were observed, but were not statistically significant. The number of documented fungal infections decreased from 12 pre-intervention (9 *Candida spp*. 3 *Cryptococcus spp*.) to 6 post-intervention (4 *Candida spp*. 2 *Cryptococcus spp*.) (*p* = 0.236). Infections caused by *Staphylococcus spp*. also decreased slightly with improved susceptibility patterns (*p* = 0.683).

**Conclusion:**

The implementation of an ASP in the critical care unit was associated with reductions in antibiotic consumption and trends toward decreased antimicrobial resistance, highlighting its potential value in addressing the challenges of antimicrobial misuse and resistance in resource-limited settings.

**Clinical trial number:**

Not applicable.

**Supplementary information:**

The online version contains supplementary material available at 10.1186/s12879-026-13358-8.

## Background

The rise of antimicrobial resistance (AMR) represents one of the most significant threats to global health. In 2019, approximately 4.95 million deaths were linked to bacterial AMR, of which 1.27 million were directly related to bacterial AMR [1]. Antimicrobial resistance has the potential to result in 10 million deaths by 2050 if the current pattern of inappropriate and excessive antibiotic use persists [2].

The overuse of antibiotics has caused multidrug-resistant organisms (MDROs) to emerge in intensive care units (ICUs), intensifying the global threat of antibiotic resistance [3–7]. As microorganisms evolve and develop resistance to existing treatments, the effectiveness of antimicrobials, such as antibiotics, antifungals, antivirals, and antiparasitics, diminishes, leading to increased morbidity, mortality, and healthcare costs [8–11].

Reducing antibiotic consumption directly impacts the development of AMR. With fewer antibiotics used, there is less selective pressure on bacteria to evolve resistance mechanisms. This is particularly important in hospitals where MDROs are a significant concern [12].

Data from the Global Antimicrobial Resistance and Use Surveillance System (GLASS) Report 2022 indicate a high consumption of antibiotics for systemic use, particularly among the access and watch groups in Egypt [13].

Many studies have evaluated antibiotic use and resistance patterns in Egypt, highlighting a high burden of antimicrobial consumption in hospital settings. A national Point Prevalence Survey (PPS) conducted across 18 Egyptian hospitals demonstrated that 59% of the 3,408 hospitalized patients received at least one antibiotic at the time of the survey. Antibiotic use was significantly higher among ICU patients and children younger than 12 years (*p* < 0.05) [14].

Additional evidence of suboptimal antimicrobial prescribing practices in Egypt was reported in another point prevalence survey conducted by Ashour et al. [15]. In this survey, 300 hospitalized patients received antimicrobial therapy, with an overall prevalence of antimicrobial prescription of 79.15%. Despite documentation of the indication for antimicrobial therapy in all cases, key stewardship components were frequently missing. A documented stop or review date was present in only 19.6% of prescriptions, and local antimicrobial guidelines were unavailable for 77.6% of cases. Furthermore, combination antimicrobial therapy was commonly prescribed (46.6% of cases), particularly in orthopedic and cardiothoracic surgical units, raising concerns about unnecessary broad-spectrum exposure and prolonged prophylaxis.

In addition to the high prevalence of antibiotic use, several studies have documented alarming rates of antimicrobial resistance among Gram-negative pathogens in Egypt. A literature review conducted by El-Kholy et al. [16] reported a wide range of resistance patterns, reflecting substantial inter-hospital variability. Extended-spectrum β-lactamase (ESBL)-producing *Enterobacteriaceae* were highly prevalent, with ESBL positivity detected in 19–85.24% of *Escherichia coli* and 10–87% of *Klebsiella pneumoniae* isolates. Even more concerning, the reported rates of carbapenem-resistant *Enterobacteriaceae* (CRE), with resistance observed in 35–100% of *K. pneumoniae* and 13.8–100% of *E. coli* isolates. These findings indicate a substantial burden of multidrug-resistant (MDR) Gram-negative organisms, particularly in critical care settings.

Further evidence of the growing burden of antimicrobial resistance in Egyptian critical care settings was reported in a study conducted by Kotb et al. [17]. The study identified 1,598 healthcare-associated infection (HAI) cases caused by *Enterobacteriaceae*, of which 871 cases (54.1%) were carbapenem-resistant, a temporal trend analysis between 2011 and 2017 revealed a significant increase in the proportion of *Enterobacteriaceae* HAI cases attributable to CRE (*p* = 0.003), along with an upward trend in the incidence of CRE-associated HAIs (*p* = 0.09).

Antimicrobial resistance represents a major public health threat in Egypt and has been associated with substantial morbidity and mortality. National and global burden estimates indicate that more than 20,000 deaths have occurred annually in Egypt since 1990 as a result of AMR. In 2021, it was estimated that 15,500 deaths (uncertainty interval [UI]: 12,500– 18,500) were directly attributable to AMR, while 52,500 deaths (UI: 42,600– 62,400) were associated with AMR. The burden of AMR-related mortality was highest among individuals aged ≥ 70 years, reflecting increased vulnerability in older populations. Furthermore, the most lethal pathogen–drug resistance combinations reported in Egypt in 2021 included methicillin-resistant *Staphylococcus aureus* (MRSA), carbapenem-resistant *Acinetobacter baumannii*, and carbapenem-resistant *Streptococcus pneumoniae*. These pathogens are frequently implicated in severe healthcare-associated infections, particularly in intensive care settings, and contribute significantly to prolonged hospital stays, increased healthcare costs, and excess mortality [18].

In response to this escalating crisis, ASPs have emerged as a critical strategy to optimize the use of antimicrobials by increasing adherence to antimicrobial guidelines. They are also recognized for improving patient care and having a positive impact on AMR, ensuring that patients receive the most appropriate treatment while minimizing the potential for resistance development [19].

ASPs encompass a broad range of interventions designed to promote the judicious use of antimicrobials across healthcare settings. These initiatives include the implementation of evidence-based prescribing guidelines, post prescription reviews (time-outs) which involves reassessing antimicrobial therapy after it has been initiated, usually at 48–72 hours, when more clinical and microbiological data become available, IV-to-oral switches, the promotion of diagnostic stewardship, antimicrobial usage monitoring, and education of healthcare providers and patients about the risks of inappropriate antimicrobial use. By improving the selection, dosing, route, and duration of antimicrobial therapy, ASPs not only enhance patient outcomes but also contribute to the preservation of antimicrobial efficacy for future generations [20–23].

Even though the majority of antimicrobial stewardship programs are reported in high-income countries [24–26], some antibiotic stewardship interventions have begun to take place in low- and middle-income countries [27, 28]. In Egypt, few studies have evaluated the effectiveness of multidisciplinary ASPs in critical care settings [29, 30].

In Egypt, antimicrobial stewardship activities are guided by a National Action Plan against Antimicrobial Resistance, which is aligned with the WHO Global Action Plan on AMR and implemented within a One Health framework encompassing human, animal, and environmental health. At the national level, the Ministry of Health and Population (MoHP) oversees ASP implementation through the General Directorate of Infection Control and National AMR Surveillance Systems, while the Egyptian Drug Authority (EDA) issues national ASP guidelines and policies for antimicrobial use based on World Health Organization (WHO) and Centers for Disease Control and Prevention (CDC) core stewardship elements [31].

At the healthcare facility level, ASPs are implemented through a multidisciplinary Antimicrobial Stewardship (AMS) committee. Committee membership typically includes a healthcare facility administrator (executive sponsor or chair), director of medical services, an AMS-trained pharmacist or physician (secretary), infectious diseases physician (if available), clinical and hospital pharmacists, microbiology representatives, nursing representatives, patient safety and clinical quality managers, medical staff representatives from different wards (particularly ICUs), infection prevention and control (IPC) committee representatives, drug and therapeutics committee representatives, and information technology personnel when available. Additional members may be co-opted as required to support stewardship activities.

ASP implementation in Egyptian hospitals commonly involves prospective audit and feedback, preauthorization or restriction of selected antimicrobials, and the use of standard treatment guidelines adapted from national or international recommendations.

AMS teams in active programs conduct regular ward rounds and antimicrobial reviews, particularly in high-risk areas such as intensive care units, with feedback provided directly to prescribers. Antibiotic use audits and prescription reviews are performed on a weekly or continuous basis in many institutions, while antimicrobial consumption reporting and resistance trend analysis are typically conducted monthly, quarterly, or biannually, depending on institutional resources. Facility-specific antibiograms are usually updated annually or biannually, where microbiology capacity allows.

Evidence from a national cross-sectional study by Hamdy et al. [32] highlights the current status of ASP implementation in Egypt. In this study, 61.8% of hospitals had adopted an ASP, while 38.2% had not, although 71.4% of the latter planned future implementation. Facility-specific antibiograms were routinely used in 48.5% of hospitals, and 37.1% produced regular antimicrobial utilization reports.

Additionally, 42.9% reported using computerized applications to support stewardship activities. Outcome indicators such as resistance patterns, infection rates, length of hospital stay, mortality, and adverse drug reactions were monitored. However, only 20% of hospitals had a formal ASP training program, indicating an important gap in workforce capacity and education.

The components of ASPs often differ among institutions because of variations in the available resources, infrastructure, and specific antimicrobial resistance challenges faced by each facility. Among the many stewardship strategies described in the literature, prospective audits and feedback, and preauthorization of antimicrobial agents have consistently been identified as the most effective interventions for reducing inappropriate antibiotic use and combating resistance [22, 33–37]. These approaches allow real-time oversight of prescribing practices, early intervention to optimize therapy, and prevention of unnecessary broad-spectrum antibiotic use. In our ASP, we prioritized these two core strategies because they offer direct and timely control over antimicrobial use while promoting prescriber accountability and education. Additionally, we ensured adherence to our locally developed guidelines, which were based on local resistance patterns and the best available evidence. This combined approach enabled us to tailor antimicrobial therapy to our institutional needs, enhance patient outcomes, and slow the emergence of resistant organisms.

This study aimed to evaluate the impact of an ASP implemented in a tertiary cardiac center in Egypt. The primary objectives were to assess changes in antimicrobial consumption, quantified using antibiotic utilization metrics, and to evaluate trends in antimicrobial resistance, including changes in susceptibility patterns and the incidence of infections caused by MDROs.

## Materials and methods

### Study design and setting

A quasi-experimental study design was used to assess the impact of the antimicrobial stewardship program on antimicrobial consumption, estimated by defined daily dose (DDD) per 1,000 patient days, and resistance patterns among adult intensive care unit patients at the Aswan Heart Center (Magdi Yacoub Foundation) from July 2023 to 30 June 2024. Although this design is appropriate for exploratory assessment, it does not fully account for potential confounding factors. In particular, seasonal variation, temporal changes in ICU clinical practices, and concurrent infection prevention and control interventions may have influenced the observed outcomes and should be considered when interpreting the findings.

The Aswan Heart Centre is a 100-bed tertiary referral hospital that provides comprehensive cardiovascular services, including percutaneous coronary intervention (PCI), cardiac surgery, and medical management of complex cardiac conditions. The hospital treats patients with medical conditions such as infective endocarditis, heart failure, arrhythmias, pulmonary hypertension, and other advanced cardiovascular diseases, in addition to interventional and surgical care. However, the majority of admissions are related to cardiac surgical procedures, including valve surgeries, coronary artery bypass grafting, and congenital heart disease repair.

All positive microbiological culture results, including blood, urine, sputum, and swab cultures, were collected during the study period. Cultures reported as contaminants or colonization were excluded according to laboratory protocols. Antimicrobial consumption data included all systemic antibacterial and antifungal agents administered via intravenous and oral routes. Data were collected for six months before and six months after the implementation of the antimicrobial stewardship program.

All adult patients admitted to the intensive care unit during the study period were included in the study. A total of 1,982 patients were included in the analysis, with 1,000 patients in the pre-intervention period and 982 patients in the post-intervention period.

The primary indications for antimicrobial therapy included treatment of suspected or confirmed infections, such as bloodstream infections, respiratory tract infections, urinary tract infections, surgical site infections, and Infective Endocarditis.

Antimicrobial consumption was defined using the WHO Anatomical Therapeutic Chemical (ATC)/Defined Daily Dose (DDD) methodology and expressed as DDD per 1,000 patient-days, which reflects the average daily dose of an antimicrobial agent consumed per 1,000 patient-days.

Antimicrobial resistance was defined as the proportion of isolates classified as resistant or non-susceptible to a given antimicrobial agent according to the Clinical and Laboratory Standards Institute (CLSI) breakpoints, excluding contaminants or colonizing isolates.

Culture results were extracted retrospectively from the hospital’s laboratory information system (LIS) and electronic patient records. All positive cultures obtained from adult ICU patients, including blood, urine, sputum, and swab samples, were included, while cultures reported as contaminants or colonization were excluded. Extracted data included patient identifier, specimen type, date of collection, microorganism identified, and antimicrobial susceptibility test results.

### Inclusion criteria

All patients who were admitted to the adult ICU from July 2023 to 30 June 2024 were included in the study. We divided the patients into two groups. The first group included all patients admitted to the ICU before ASP implementation (from July 1, 2023, to December 31, 2023). The second group comprised all patients admitted to the ICU after the implementation of the ASP (from January 1, 2024, to June 30, 2024).

Antimicrobial consumption data included all systemic antimicrobial agents, encompassing both intravenous and oral formulations. Both antibacterial and antifungal agents were included in the analysis in order to capture total antimicrobial use in the ICU.

The following major antibiotic classes were included: β-lactams (Penicillins, Cephalosporins, Carbapenems), Glycopeptides, Aminoglycosides, Fluoroquinolones, Oxazolidine, Trimethoprim–sulfamethoxazole, Polymyxins, Tetracyclines, Lincosamides, and systemic antifungal agents.

Microbiological analysis included clinically significant bacterial and fungal isolates recovered from patients during the study period. The microorganisms of interest comprised *Enterobacteriaceae*, including *Enterobacter spp.*, *Klebsiella spp.*, *Proteus spp.*, *Escherichia coli*., and *Serratia spp.* In addition, Gram-positive bacteria such as *Staphylococcus spp.* and fungal pathogens, including *Candida spp.* and *Cryptococcus spp.*, were included.

### Exclusion criteria

Pediatric or neonatal patients were excluded from this study. In addition, isolates considered to represent microbiological contamination rather than true infection, as determined by clinical assessment and microbiological criteria, were excluded from the analysis. Furthermore, duplicate isolates from the same patient were excluded during antimicrobial resistance analysis and antibiogram generation using WHO Global Antimicrobial Resistance Surveillance System (WHONET).

### Data collection

We used pharmacy dispensing records to calculate the DDD for antimicrobial consumption, and laboratory culture results to perform antibiograms and estimate antibacterial resistance. Antimicrobial resistance was assessed using microbiological culture and susceptibility testing performed in the hospital laboratory. All bacterial and fungal isolates obtained from clinically relevant specimens (blood, urine, sputum, and swabs) were included, while contaminants and colonizing isolates were excluded. Clinically significant isolates were defined based on standard laboratory criteria in conjunction with clinical correlation; isolates were considered significant when recovered from sterile sites or when associated with documented clinical signs and symptoms of infection. In contrast, isolates deemed contaminants (e.g., common skin flora isolated from a single blood culture without clinical evidence of infection) or colonizers (i.e., organisms isolated in the absence of clinical infection) were excluded in accordance with established laboratory protocols and infection control guidelines. Microbial identification and susceptibility testing were performed using the VITEK automated system (bioMérieux, France) according to CLSI guidelines. Isolates were categorized as susceptible, intermediate, or resistant based on CLSI breakpoints. Resistance rates were calculated as the proportion of isolates classified as resistant or non-susceptible to each antimicrobial agent. Data were analyzed using the WHONET. To ensure accuracy and avoid bias, duplicate isolates from the same patient were excluded during antimicrobial resistance analysis and antibiogram generation, whereby only the first isolate per patient for each organism was included.

Antimicrobial consumption was measured using the WHO Anatomical Therapeutic Chemical/Defined Daily Dose (ATC/DDD) methodology, which is the recommended standard for antimicrobial stewardship studies. Total consumption of each antimicrobial agent was calculated by dividing the total amount of drug used by the WHO-assigned DDD. Consumption was expressed as DDD per 1,000 patient-days, providing a standardized measure that allows comparison across time periods and facilities. Both intravenous and oral systemic agents were included, encompassing antibacterial and antifungal agents. We used the ICU data collection registry to extract patient demographics and characteristics for the two groups.

### Outcome measures

The primary outcomes were total systemic antimicrobial consumption in the ICU, which was calculated as the DDD per 1,000 patient days per month; the incidence and antimicrobial resistance patterns of *Enterobacteriaceae*, the predominant cause of bacterial infections in our hospital; and the incidence and antifungal resistance rates of fungal infections, as well as the incidence and resistance patterns of *Staphylococcus* infections.

Monthly antimicrobial consumption was calculated by dividing the total number of DDDs used each month by the total number of occupied bed-days in the same month and multiplying by 1,000. Thus, the unit of analysis was defined as DDD per 1,000 patient-days per month. The monthly DDD values were then summed over six consecutive months to estimate the total antimicrobial consumption in the pre-intervention and post-intervention periods, and these aggregated values were used for comparisons between the two periods. Presenting data per month allows assessment of temporal trends in antimicrobial use before and after implementation of the stewardship program. Reporting on a monthly basis captures fluctuations in consumption and facilitates evaluation of the intervention’s immediate and sustained impact.

### Statistical analysis

Simple descriptive statistics were used to present the sample’s demographic characteristics. Baseline demographic and clinical characteristics were summarized for all included patients. For a small number of missing values (5 patients in the pre-intervention period and 2 patients in the post-intervention period), data were excluded only from analyses of the specific variable with missing information. The affected patients were retained in all other analyses for which complete data were available. Categorical variables are represented in the form of frequencies and percentages, whereas continuous variables are expressed as the means and standard deviations.

Continuous variables were assessed for normality prior to analysis using the Shapiro–Wilk test. Comparisons between the pre-intervention and post-intervention groups were performed using the independent (Student’s) t-test for normally distributed continuous data and the Mann–Whitney U test for non-normally distributed data. Categorical variables were compared using Fisher’s exact test.

The Jamovi Project (2024). Jamovi (Version 2.5) [Computer software]. Available from: https://www.jamovi.org was used for data analysis. A *p*-value < 0.05 was considered statistically significant.

### Antimicrobial stewardship components

A structured antimicrobial stewardship program was implemented starting in January 2024 to improve antimicrobial prescribing practices and reduce antibiotic resistance in the ICU. The AMS program described in this study was newly implemented during the study period. It was introduced as a hospital-wide quality improvement initiative in response to the markedly increasing rates of antimicrobial resistance observed in recent years. The AMS program was developed in accordance with the CDC Core Elements of Hospital Antimicrobial Stewardship Programs, with adaptation to local hospital needs, patient populations, and antimicrobial resistance patterns.

The program began with the establishment of an Antimicrobial Stewardship Committee, composed of a multidisciplinary team including an infectious disease–trained physician (program leader), a clinical pharmacist (co-leader), infection control specialist, quality specialist, information technology representative, heads of clinical departments, microbiologists, nurses, and surgeons. The committee holds regular quarterly meetings to review antimicrobial utilization, resistance trends, stewardship performance, and to guide the implementation of interventions. Initial meetings were conducted to define stewardship priorities and agree on the interventions to be implemented hospital-wide.

The components of the antimicrobial stewardship program in our hospital included the following, several of which were newly implemented during the study period:

1. Preauthorization of restricted antimicrobials (newly implemented)
  Preauthorization was required before prescribing selected restricted antimicrobials, including colistin, tigecycline, linezolid, ceftazidime/avibactam, daptomycin, ceftaroline, and meropenem. This applied continuously for all eligible prescriptions and approval provided by the AMS team prior to initiation.
2. Development of local antimicrobial treatment protocols.
  An Antibiotic protocol booklet was developed based on local susceptibility data to guide empirical antibacterial therapy and was updated annually according to the hospital antibiogram. Included a dedicated protocol for preoperative surgical prophylaxis.
3. Prospective audit and feedback
  Prospective audits and feedback were performed by clinical pharmacists, who reviewed antimicrobial therapy regarding indication, drug selection, dose, route, and duration. This conducted daily after antimicrobial prescription and recommendations were communicated directly to prescribing clinicians
4. Use of procalcitonin and rapid diagnostic testing (newly implemented)
  The program encouraged the use of procalcitonin testing to support decisions on antimicrobial de- escalation and discontinuation. In addition, the program promoted the adoption of rapid molecular diagnostic tools (BioFire® FilmArray® Panels) for early pathogen identification and detection of resistance genes. This used when clinically indicated, particularly in critically ill patients and aimed to enable targeted therapy and reduce unnecessary broad-spectrum antimicrobial exposure.
5. Development of treatment algorithms (newly implemented).
  Empirical prescribing was further optimized through the development of simplified treatment algorithms for common bacterial infections at our institution (e.g., surgical site infections, hospital-acquired pneumonia, community-acquired infection, bloodstream infections). Specific guidance for multidrug-resistant organisms based on international evidence-based guidelines, including the Infectious Diseases Society of America (IDSA) guidelines, and were adapted to local practice by incorporating local antibiogram data, and patient- specific risk factors for multidrug-resistant organisms (e.g., prior antibiotic exposure, prolonged ICU stay, recent hospitalization). Detailed examples of empiric treatment algorithms are provided in Supplementary Documents (1–6).
6. Additional stewardship interventions
  Additional components of the program included: IV-to-oral switch strategies, evaluated daily in eligible patients. Regular education and training sessions for healthcare providers on appropriate antimicrobial use, delivered on an ongoing basis.

### Ethical consideration

Ethical approval was obtained from the Institutional Review Board (IRB) at Aswan Heart Centre (Aswan Heart Centre Research Ethics Committee AHC-REC). REC Approval number: 20241225MYFAHC_ASP_20250213. As the study imposed no risk to the subjects, and the data were aggregated and anonymized in the reporting of findings, the Aswan Heart Centre Research Ethics Committee waived the need to have an informed signed consent form (ICF) from the participants in our study.

## Results

### Baseline characteristics

During the pre-intervention phase, 1000 patients were admitted to the ICU, whereas during the post-intervention period, 982 patients were admitted to the ICU. There were no significant differences in baseline characteristics, including demographics, ICU mortality rates, or diagnoses on admission to the ICU, except for age (Table 1).

**Table 1:** Baseline demographic and clinical characteristics of the study participants before and after ASP implementation

Variable	Pre-intervention (*n* = 1000)	Post-intervention (*n* = 982)	*p* -value
Demographic characteristics			
Male, n (%)	577 (57.7%)	536 (54.6%)	0.174
Female, n (%)	423 (42.3%)	446 (45.4%)	0.174
Age, mean (SD)	47.3 (16.5)	49.1 (17.2)	0.018
Mortality, n (%)	27 (2.7%)	29 (3.0%)	0.787
Type of ICU admission			
Surgery, n (%)	317 (31.7%)	283 (28.8%)	0.171
Medical treatment, n (%)	90 (9.0%)	84 (8.6%)	0.751
Coronary interventions, n (%)	267 (26.7%)	281 (28.6%)	0.366
Structural heart interventions, n (%)	84 (8.4%)	72 (7.3%)	0.405
Electrophysiology, n (%)	108 (10.8%)	104 (10.6%)	0.885
Others, n (%)	129 (12.9%)	156 (15.9%)	0.063
Missing data, n (%)	5 (0.5%)	2 (0.2%)	0.458
Type of cultures			
Blood, n (%)	35 (25%)	17 (20%)	0.420
Sputum, n (%)	47 (33%)	22 (26%)	0.239
Swab, n (%)	23 (16%)	20 (23%)	0.222
Urine, n (%)	37 (26%)	27 (31%)	0.45

ASP, antimicrobial stewardship programme; ICU, intensive care unit; SD, standard deviation, Mortality refers to all-cause mortality during the index hospitalization

### Antimicrobial consumption

The total monthly antimicrobial utilization decreased by 23%, from 14,414 to 11,012 DDD per 1,000 patient days following ASP implementation; however, this reduction did not reach statistical significance (*p* = 0.097). A statistically significant decrease was observed in colistin consumption, which declined by 764 DDD per 1,000 patient days, from 817 to 53 (*p* = 0.006). Reductions were also noted in the use of tigecycline and ceftazidime-avibactam, with mean decreases of 36 and 136 DDD per 1,000 patient days, respectively; however, these changes were not statistically significant (*p* = 0.161 and *p* = 0.100, respectively).

In contrast, meropenem consumption increased slightly by 89 DDD per 1,000 patient days (from 1,169 to 1,258). Similarly, increases were observed in levofloxacin, trimethoprim-sulfamethoxazole, and linezolid use, with mean increases of 59, 147, and 12 DDD per 1,000 patient days, respectively; however, none of these changes reached statistical significance (*p* = 0.905, *p* = 0.979, and *p* = 0.739, respectively). These variations in individual antimicrobial use may reflect shifts in prescribing practices following ASP implementation, such as increased reliance on culture-guided therapy, de-escalation from more toxic or restricted agents, and optimization of therapy through intravenous-to-oral switching when clinically appropriate. They may also be influenced by differences in patient case-mix and infection severity during the study periods. Overall, these findings suggest evolving prescribing patterns rather than a uniform reduction across all antimicrobial classes (Tables 2, 3).

**Table 2:** Comparison of mean systemic antimicrobial consumption (DDD per 1,000 patient days) at Aswan Heart center during pre-intervention (Jul – Dec 2023) and post-intervention (Jan–Jun 2024) periods of the antimicrobial stewardship program implementation

Antimicrobial Agent	Pre-intervention Mean ± SD	Post-intervention Mean ± SD	*p*-value
Amikacin	21.9 ± (37.9)	0 ± (0)	0.093
Ampicillin-sulbactam	11.2 ± (9.12)	15.6 ± (21.8)	0.670
Amoxicillin-clavulanic acid (oral)	45.5 ± (48)	35.5 ± (20.6)	0.324
Liposomal Amphotericin	119 ± (150)	0 ± (0)	0.040
Cefazolin	469 ± (168)	472 ± (122)	0.515
Cefepime	39 ± (35.1)	32.8 ± (24.2)	0.364
Linezolid (oral, IV)	57 ± (32.8)	69.4 ± (31.8)	0.739
Levofloxacin (oral, IV)	53.2 ± (18.2)	111 ± (101)	0.905
Meropenem	195 ± (136)	210 ± (86.2)	0.588
Piperacillin-tazobactam	113 ± (59.6)	113 ± (34.4)	0.499
Tigecycline	81.5 ± (72.4)	45.9 ± (41.6)	0.161
Vancomycin	261 ± (67.9)	269 ± (41.1)	0.593
Trimethoprim-sulfamethoxazole (oral)	96.1 ± (40.5)	216 ± (150)	0.979
Ceftaroline	16 ± (25.7)	0 ± (0)	0.079

ASP, antimicrobial stewardship programme; DDD, defined daily dose; SD, standard deviation

**Table 3:** Comparison of median systemic antimicrobial consumption (DDD per 1,000 bed days) at Aswan Heart center during pre-intervention (Jul– Dec 2023) and post-intervention (Jan–Jun 2024) periods of the antimicrobial stewardship program implementation

Antimicrobial	Pre-intervention Median (IQR)	Post-intervention Median (IQR)	p-value
Caspofungin	24 (20–75)	5 (0–19)	0.096
Ceftazidime	18 (3–44)	53 (41–69)	0.853
Ceftazidime-Avibactam	12 (2.31–25.5)	0 (0–2.54)	0.100
Ceftriaxone	21 (7–41)	3 (0–11)	0.071
Ciprofloxacin (oral)	4 (1–8)	0.8 (0–2)	0.140
Ciprofloxacin (IV)	0.5 (0–6)	3 (0.5–5)	0.719
Clindamycin (IV)	9 (2–57)	6 (3–9)	0.286
Colistin	50 (36–221)	0 (0–14)	0.006
Fluconazole (oral)	1 (0–2)	3 (0.7–6)	0.819
Fluconazole (IV)	11 (2–23)	3 (0.5–20)	0.403
Gentamicin	0 (0–1)	28 (24–34)	0.999
Metronidazole (oral)	29 (27–32)	26 (10–40)	0.531
Voriconazole (oral, IV)	4 (0.5–8)	12 (5–60)	0.887
Azithromycin	16 (3–21)	0 (0–4)	0.174
Teicoplanin	431 (107–507)	0 (0–14)	0.052

ASP, antimicrobial stewardship programme; DDD, defined daily dose; IQR, interquartile range

### Antimicrobial resistance

There was a significant reduction in infections caused by *Klebsiella pneumoniae*, decreasing from 42 isolates in the pre-intervention period to 19 isolates in the post-intervention period (*p* = 0.004), representing a marked decline in one of the most problematic multidrug-resistant pathogens in the study setting.

Regarding susceptibility patterns among *Enterobacteriaceae*, post-intervention isolates demonstrated overall improvement in susceptibility to several key antimicrobial classes, particularly aminoglycosides and carbapenems in *Escherichia coli*, susceptibility to gentamicin increased from 36% (4/11 isolates) in the pre-intervention period to 86% (12/14 isolates) post-intervention (*p* = 0.01), while susceptibility to meropenem increased from 64% (7/11 isolates) to 100% (14/14 isolates) (*p* = 0.02).

For *Klebsiella pneumoniae*, although susceptibility percentages to some antimicrobials remained relatively stable, the absolute reduction in the number of isolates suggests a decreased burden of infection rather than a shift toward increased resistance. Tigecycline maintained high activity against *K. pneumoniae* in both periods, with susceptibility rates of 97% (41/42 isolates) in the pre-intervention period and 100% (19/19 isolates) post-intervention.

Following the intervention, a reduction was observed in both the incidence of fungal infections and antifungal susceptibility patterns. Before the intervention, a total of 12 fungal infections were documented, including 9 cases caused by *Candida* species and 3 cases caused by *Cryptococcus* species. After the intervention, the overall incidence of fungal infections decreased to 6 cases, with 4 cases attributed to *Candida* species and 2 cases caused by *Cryptococcus* species; however, this reduction did not reach statistical significance (*p* = 0.236). Among *Candida* isolates, susceptibility to fluconazole increased from 56% (5/9 isolates tested) to 100% (4/4 isolates), and susceptibility to amphotericin B improved from 56% (5/9 isolates) to 100% (4/4 isolates), although these changes were not statistically significant (*p* = 0.22 for both). Similarly, susceptibility to caspofungin increased from 80% (7/9 isolates) to 100% (4/4 isolates), which was also not statistically significant (*p* = 1.00). These findings suggest improved antifungal susceptibility; however, they should be interpreted cautiously given the small number of isolates.

The number of infections caused by *Staphylococcus* species decreased from 13 cases in the pre-intervention period to 11 cases in the post-intervention period. This was accompanied by numerical increases in susceptibility to several antimicrobial agents, including clindamycin (75% [10/13] vs. 100% [11/11]), ciprofloxacin (62% [8/13] vs. 81% [9/11]), gentamicin (39% [5/13] vs. 64% [7/11]), and trimethoprim–sulfamethoxazole (69% [9/13] vs. 91% [10/11]); however, these changes were not statistically significant and should be interpreted with caution.

Antimicrobial susceptibility results of the isolated pathogens were interpreted according to CLSI guidelines. Table 4 shows the changes in antimicrobial susceptibility patterns before and after the intervention for the most clinically relevant Gram-negative, Gram-positive, and fungal organisms, including the number of isolates for each microbial species and their susceptibility to the tested antimicrobial agents. Detailed susceptibility results for all isolates are provided in the Supplementary Materials (Table S1).

**Table 4:** Antimicrobial susceptibility of key bacterial isolates at Aswan Heart center during pre-intervention (Jul – Dec 2023) and post-intervention (Jan–Jun 2024) periods of the antimicrobial stewardship program implementation

Microbial species	No. of isolates (Pre)	No. of isolates (Post)	Antimicrobial	Susceptibility Pre-intervention n (%)	Susceptibility Post-intervention n (%)	*p*-value
Gram-negative Bacteria						
*Escherichia coli*	**11**	**14**	**Amikacin**	9/11 (82)	13/14 (93)	0.56
			**Cefepime**	4/11 (36)	3/14(21)	0.65
			**Gentamicin**	4/11 (36)	12/14 (86)	0.01*
			**Levofloxacin**	3/11 (27)	9/14 (64)	0.1
			**Meropenem**	7/11 (64)	14/14 (100)	0.02*
			**Piperacillin/Tazobactam**	5/11 (46)	10/14 (71)	0.2
			**Tigecycline**	11/11 (100)	14/14 (100)	
			**Trimethoprim/Sulfamethoxazole**	2/11 (18)	4/14 (29)	0.66
*Klebsiella pneumoniae*	**42**	**19**	**Amikacin**	25/42 (59)	11/19 (58)	1
			**Cefepime**	7/42 (17)	7/19(37)	0.106
			**Gentamicin**	20/42 (48)	8/19 (42)	0.78
			**Levofloxacin**	19/42 (46)	11/19 (59)	0.41
			**Meropenem**	21/42 (50)	7/19 (37)	0.41
			**Piperacillin/Tazobactam**	15/42 (36)	9/19 (47)	0.41
			**Tigecycline**	41/42 (97)	19/19 (100)	1
			**Trimethoprim/Sulfamethoxazole**	17/42 (41)	7/19 (37)	1
Gram-positive Bacteria						
*Staphylococcus aureus*	13	11	**Ampicillin**	2/13 (15)	2/11 (18)	1
			**Ciprofloxacin**	8/13 (62)	9/11(81)	0.38
			**Clindamycin**	10/13 (75)	11/11(100)	0.2
			**Gentamicin**	5/13 (39)	7/11 (64)	0.41
			**Levofloxacin**	9/13 (69)	9/11 (82)	0.64
			**Moxifloxacin**	7/13 (50)	11/11 (100)	0.01*
			**Rifampicin**	12/13 (92)	11/11 (100)	1
			**Trimethoprim/** **Sulfamethoxazole**	9/13 (69)	10/11 (91)	0.32
Fungal Isolates						
*Candida spp.*	**9**	**4**	**Voriconazole**	9/9 (100)	4/4 (100)	
			**Amphotericin B**	5/9 (56)	4/4 (100)	0.22
			**Fluconazole**	5/9 (56)	4/4 (100)	0.22
			**Caspofungin**	7/9 (80)	4/4 (100)	1
*Cryptococcus spp.*	**3**	**2**	**Voriconazole**		2/2 (100)	
			**Amphotericin B**	3/3 (100)	2/2 (100)	
			**Fluconazole**		2/2 (100)	

Data are presented as number of susceptible isolates over total tested isolates with corresponding percentages. *p*-values were calculated using Fisher’s exact test

*Statistically significant difference (*p* < 0.05)

## Discussion

This study demonstrated that the implementation of an ASP was associated with a reduction in overall antimicrobial consumption. particularly broad-spectrum agents. By promoting the appropriate use of narrow-spectrum antimicrobials whenever feasible, ASPs help preserve the effectiveness of these agents.

In our study, there was an overall 23% reduction in antimicrobial consumption in terms of DDDs after ASP implementation (from 14,414 per 1,000 patient days to 11,012 per 1,000 patient days); however, this reduction did not reach statistical significance (*p* = 0.097). A statistically significant reduction was observed in the consumption of colistin, which is primarily used for the treatment of multidrug-resistant Gram-negative infections.

Changes in antimicrobial use were accompanied by shifts in microbiological outcomes. A statistically significant decrease was observed in infections caused by *Klebsiella pneumoniae*. In addition, a significant improvement in susceptibility of *Escherichia coli* to meropenem and gentamicin was noted.

There was also a reduction in the incidence of fungal infections, antifungal resistance rates, and the incidence and resistance patterns of *Staphylococcus* spp.; however, these changes did not reach statistical significance, which may be explained by the limited number of isolates.

It is important to consider that factors other than the ASP may have contributed to the observed findings. Potential changes in infection prevention and control practices, variations in patient case mix, and differences in disease severity between the pre- and post-intervention periods may have influenced antimicrobial use, infection rates, and resistance patterns. Although no major institutional infection control policy changes were formally implemented during the study period, unmeasured factors—such as variability in adherence to infection control measures, staffing fluctuations, and differences in patient severity—may have influenced the results. Therefore, these factors should be taken into account when interpreting the results, and the observed associations cannot be attributed solely to the ASP.

A statistically significant difference in age was also observed between the two groups; however, the absolute difference was small and is unlikely to be clinically meaningful. Nevertheless, age may influence infection risk, antimicrobial utilization, and clinical outcomes, and therefore its potential role as a confounding factor cannot be fully excluded when interpreting the observed effects of the antimicrobial stewardship program.

Meropenem consumption demonstrated a modest increase relative to antibiotics used to treat carbapenem-resistant *Enterobacteriaceae*, such as ceftazidime/avibactam, colistin, and tigecycline. This increase in meropenem usage may have resulted from a decrease in the percentage of carbapenem-resistant *Enterobacteriaceae*. During the pre-intervention period, when the rate of CRE was high, infections caused by these organisms were managed using alternative agents with retained activity, such as ceftazidime–avibactam, colistin, and tigecycline. Following implementation of the antimicrobial stewardship program, a significant reduction in CRE rates was observed, accompanied by an increase in meropenem susceptibility among *Enterobacteriaceae* isolates. Notably, the sensitivity of *Escherichia coli* to meropenem increased from 64% to 100%, and the sensitivity of *Proteus* species increased from 67% to 100%. These improvements likely influenced prescribing practices, leading clinicians to preferentially use meropenem as a targeted and effective therapy for infections caused by these organisms. Therefore, the observed increase in meropenem consumption reflects a shift toward appropriate, culture-guided use of meropenem for carbapenem-susceptible organisms, rather than increased empirical reliance on broader-spectrum or last-resort agents.

The use of levofloxacin, trimethoprim-sulfamethoxazole, and linezolid increased slightly, possibly due to the increased rate of IV-to-oral switching, although the increase was not significant. The findings of our study are consistent with previously published evidence demonstrating the effectiveness of ASPs in reducing antimicrobial consumption and improving resistance patterns. A recent systematic review and meta-analysis published in 2023 by Zay Ya et al. which included 40 studies from high-income countries and 12 from low- and middle-income countries (LMICs), reported a 28% reduction in antibiotic consumption following ASP implementation. The same analysis also demonstrated a 28% decrease in the use of WHO Watch group antibiotics [38]. These results closely align with the reductions in overall antimicrobial consumption observed in our study, supporting the effectiveness of ASPs across diverse healthcare settings.

Comparable findings have also been reported in individual studies conducted in LMICs. A study from India conducted by Zirpe et al. showed that after ASP adoption, total DDDs per 100 patient days decreased by 18.72% (from 103.46 to 84.09 DDDs per 100 patient days), while days of therapy (DOT) were reduced by 21.62% (from 90.91 to 71.25 days) [39]. Similarly, studies from Europe and the Middle East have demonstrated meaningful reductions in antimicrobial utilization following stewardship interventions. In Italy, Fortini et al. reported a 5.3% reduction in overall antibiotic consumption, accompanied by a more than 50% decrease in carbapenem use [40]. Likewise, Mahmoudi et al. observed substantial reductions in the use of last-resort antimicrobials, including colistin, carbapenems, vancomycin, and several antifungal agents, following ASP implementation. The greatest decreases were observed for voriconazole (−47%), colistin (−35.3%), and meropenem (−39.2%) [41]. Similar trends have been observed in multicenter studies from Colombia conducted by Pallares et al., where significant reductions in the consumption of broad-spectrum agents such as meropenem, cefepime, and ceftriaxone were accompanied by improved resistance profiles. All institutions demonstrated decreased antibiotic consumption, with reductions ranging from 20% to 45%. Significant declines in meropenem, cefepime, and ceftriaxone use were also observed (*p* < 0.001), findings that are consistent with and supportive of our results. [42].

In addition to reductions in antimicrobial consumption, our study demonstrated a decrease in antimicrobial resistance rates, particularly among carbapenem-resistant *Enterobacteriaceae.* This finding is consistent with prior reports showing improvements in resistance patterns following ASP implementation. Alakkad et al. reported a marked reduction in antimicrobial resistance following stewardship interventions, with carbapenem resistance among *Enterobacter* species decreasing from 31% to 8% and quinolone resistance in *E. coli* declining from 40% to 34% [43].

Further supporting evidence comes from a retrospective observational study by Darwish et al. in two private hospitals in Jordan, which demonstrated improved bacterial susceptibility patterns and numerical reductions in *Escherichia coli* (from 425 to 356) and *Klebsiella pneumoniae* (from 140 to 88), following ASP implementation. These findings highlight the overall positive impact of stewardship programs on antimicrobial use and resistance patterns [44].

Taken together, these studies reinforce our findings and demonstrate that ASPs are effective in reducing antimicrobial consumption and mitigating antimicrobial resistance across a wide range of healthcare settings, including tertiary care hospitals in LMICs. The consistency of our results with international data underscores the value of implementing structured stewardship interventions, particularly in resource-limited settings where the burden of antimicrobial resistance is substantial.

## Limitations

This study has several limitations. First, the quasi-experimental retrospective before–after design lacks randomization and a concurrent control group, represents a major methodological limitation. Without a parallel control group, it is not possible to definitively attribute the observed changes in antimicrobial consumption and resistance patterns solely to the implementation of the ASP. Accordingly, a causal relationship cannot be established, and the findings should be interpreted as associations rather than direct effects of the intervention. In addition, temporal trends, seasonal variations, or unrelated institutional changes occurring during the study period may have influenced the results.

Second, a formal sample size calculation and power analysis were not performed prior to the study, as the analysis was based on available ICU admissions and microbiological isolates during the defined pre- and post-intervention periods. Consequently, the study was likely underpowered to detect smaller but clinically meaningful differences, particularly in antimicrobial resistance rates and among less frequently isolated pathogens.

Furthermore, the relatively small number of isolates included in certain resistance analyses may limit the reliability and precision of the observed susceptibility trends. This should be taken into account when interpreting changes in antimicrobial resistance patterns.

Third, the presence of potential confounding factors cannot be fully excluded. Changes in case mix, severity of illness, infection control practices, or staffing patterns during the study period may have contributed to the observed outcomes independently of the ASP intervention. In addition, a statistically significant difference in age between the pre- and post-intervention groups was observed. Although the magnitude of this difference was small, age is a clinically relevant factor that may influence susceptibility to infection, antimicrobial prescribing practices, and patient outcomes. Therefore, the potential impact of age as a confounding variable cannot be entirely excluded when interpreting the study results.

Fourth, antimicrobial consumption was measured using DDD per 1,000 patients- days, which, although recommended by the World Health Organization for benchmarking, may not accurately reflect actual antibiotic exposure at the individual patient level. This methodology may overestimate or underestimate true antimicrobial use in the ICU setting as DDD is based on standard adult maintenance doses, which may differ from actual prescribed doses in critically ill patients. ICU patients often receive dose adjustments due to renal impairment, hepatic dysfunction, augmented renal clearance, or severe infections requiring higher than standard dosing. DDD does not account for treatment duration. at the individual patient level. In contrast, DOT is considered a more accurate and clinically relevant metric, as it is not affected by dose modifications and better reflects real-world prescribing practices. However, implementing DOT requires patient-level, day-by-day antimicrobial administration data, which were not consistently available in our hospital’s electronic or paper-based records during the study period.

Finally, this was a single-center study conducted in one ICU, which may limit the generalizability of the findings to other hospitals, settings, or healthcare systems with different patient populations, antimicrobial prescribing practices, and stewardship resources.

## Conclusion

This study suggests that the implementation of an ASP in a critical care unit in a low-resource setting was associated with a reduction in overall antimicrobial consumption and improvements in susceptibility patterns among bacterial and fungal pathogens. A concurrent decrease in the incidence of multidrug-resistant organisms was also observed following ASP implementation.

These findings indicate that core stewardship interventions—particularly prospective audit and feedback and preauthorization of selected antimicrobial and antifungal agents—may contribute to more appropriate antimicrobial use and improved resistance patterns. However, these results should be interpreted with caution, as the quasi-experimental design, absence of a control group, and relatively small sample sizes for some analyses limit the ability to establish causality and may affect the precision of the observed estimates. Further controlled studies are warranted to better evaluate the impact of ASPs in similar settings and to confirm the generalizability of these findings.

## Electronic supplementary material

Below is the link to the electronic supplementary material.

Supplementary Material 1

Supplementary Material 2

Supplementary Material 3

Supplementary Material 4

Supplementary Material 5

Supplementary Material 6

Supplementary Material 7

Supplementary Material 8

Supplementary Material 9

Supplementary Material 10

Supplementary Material 11

Supplementary Material 12

Supplementary Material 13

Supplementary Material 14

Supplementary Material 15

Supplementary Material 16

Supplementary Material 17

Supplementary Material 18

Supplementary Material 19

Supplementary Material 20

Supplementary Material 21

## Data Availability

The datasets used and generated in this study are included in this published article and its supplementary information files

## Abbreviations

AMR: Antimicrobial resistance
ASP: Antimicrobial stewardship program
ATC: Anatomical Therapeutic Chemical
CDC: Centers for Disease Control and Prevention
CLSI: Clinical and Laboratory Standards Institute
DDD: Defined daily dose
DOT: Days of therapy
EDA: Egyptian Drug Authority
GLASS: Global Antimicrobial Resistance and Use Surveillance System
ICF: Informed consent form
ICU: Intensive care unit
IPC: Infection prevention and control
IDSA: Infectious Diseases Society of America
IRB: Institutional review board
LMICs: Low- and middle-income countries
MDROs: Multidrug-resistant organisms
MoHP: Ministry of Health and Population
WHO: World Health Organization
