# Supplementary material for: Evaluating the effects of antimicrobial stewardship program on antimicrobial consumption and resistance patterns: a quasi-experimental study
Source: BMC Infect Dis. 2026 May 20;26:988. doi: 10.1186/s12879-026-13358-8 (PMC13195955; doi:10.1186/s12879-026-13358-8)
Supplement: Supplementary file 2 — Supplementary Material 2 [file 12879_2026_13358_MOESM2_ESM.pdf]

Community-acquired pneumonia for Adult in ward

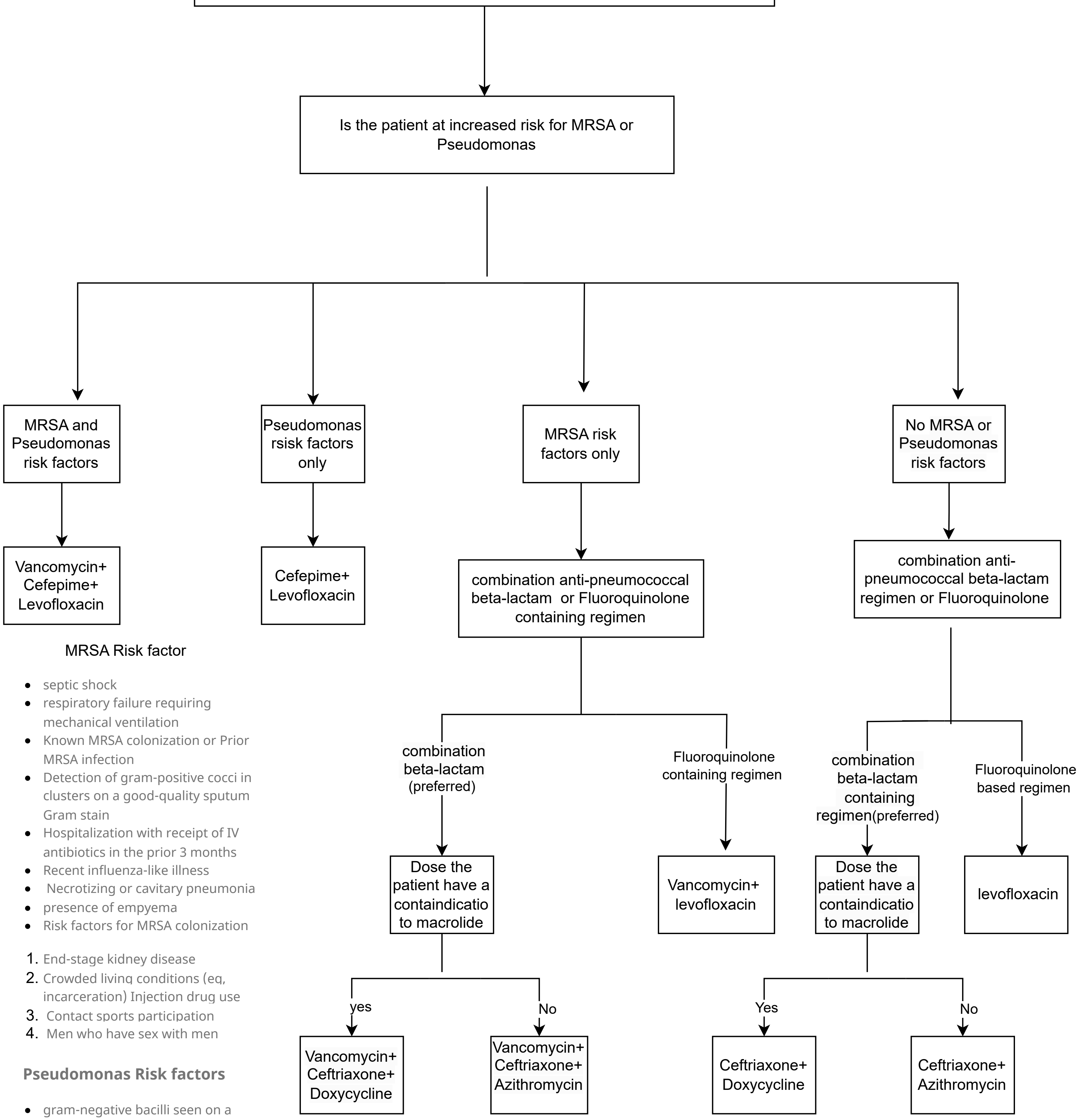

MRSA Risk factor

- septic shock
- respiratory failure requiring mechanical ventilation
- Known MRSA colonization or Prior MRSA infection
- Detection of gram-positive cocci in clusters on a good-quality sputum Gram stain
- Hospitalization with receipt of IV antibiotics in the prior 3 months
- Recent influenza-like illness
- Necrotizing or cavitary pneumonia
- presence of empyema
- Risk factors for MRSA colonization

1. End-stage kidney disease
2. Crowded living conditions (eg, incarceration) Injection drug use
3. Contact sports participation
4. Men who have sex with men

Pseudomonas Risk factors

- gram-negative bacilli seen on a good-quality sputum Gram stain
- Known colonization or infection with pseudomonas
- Hospitalization with receipt of IV antibiotics in the prior 3 months
- structural lung abnormalities (eg, bronchiectasis)
- frequent COPD exacerbation requiring frequent glucocorticoid or antibiotic use
