# Supplementary material for: Evaluating the effects of antimicrobial stewardship program on antimicrobial consumption and resistance patterns: a quasi-experimental study
Source: BMC Infect Dis. 2026 May 20;26:988. doi: 10.1186/s12879-026-13358-8 (PMC13195955; doi:10.1186/s12879-026-13358-8)
Supplement: Supplementary file 3 — Supplementary Material 3 [file 12879_2026_13358_MOESM3_ESM.pdf]

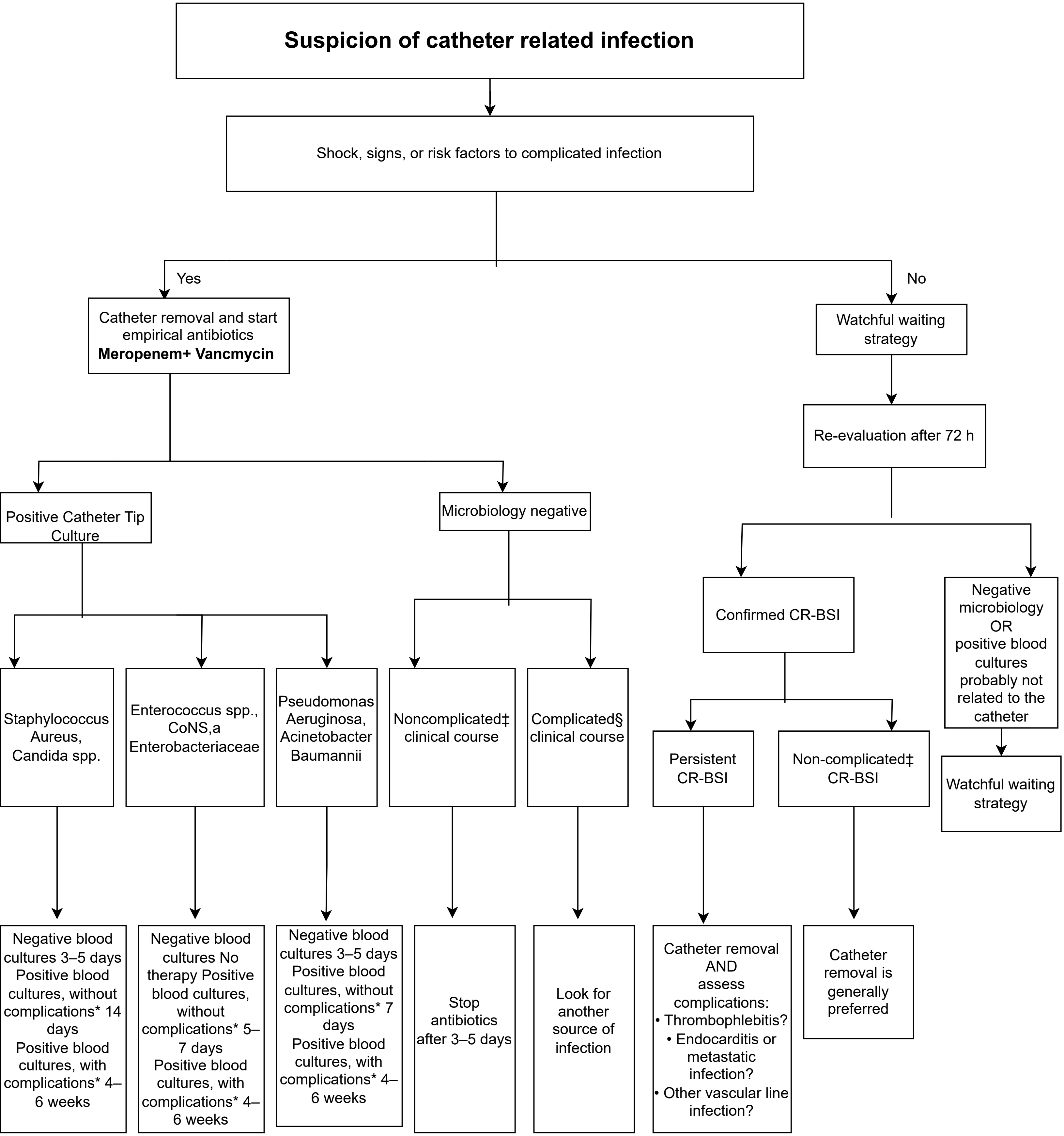

‡Favorable clinical course without persistence of fever and negative blood cultures after 72 hours of adequate treatment; and no septic metastasis, endocarditis, or septic thrombophlebitis; and without other intravascular devices or immunosuppression.

§Persistence of fever or inflammatory signs after 72 hours or septic metastasis, endocarditis, or septic thrombophlebitis

*Hemodynamic instability.Neutropenia (,500/mm) or immunosuppression (including organ transplantation). Local exit site signs (purulent discharge or redness/cellulitis 0.5 cm diameter)
