# Supplementary figures and images for: Evaluating the effects of antimicrobial stewardship program on antimicrobial consumption and resistance patterns: a quasi-experimental study
Source: BMC Infect Dis. 2026 May 20;26:988. doi: 10.1186/s12879-026-13358-8 (PMC13195955; doi:10.1186/s12879-026-13358-8)

# Suspected Hospital Aquired Pneumonia

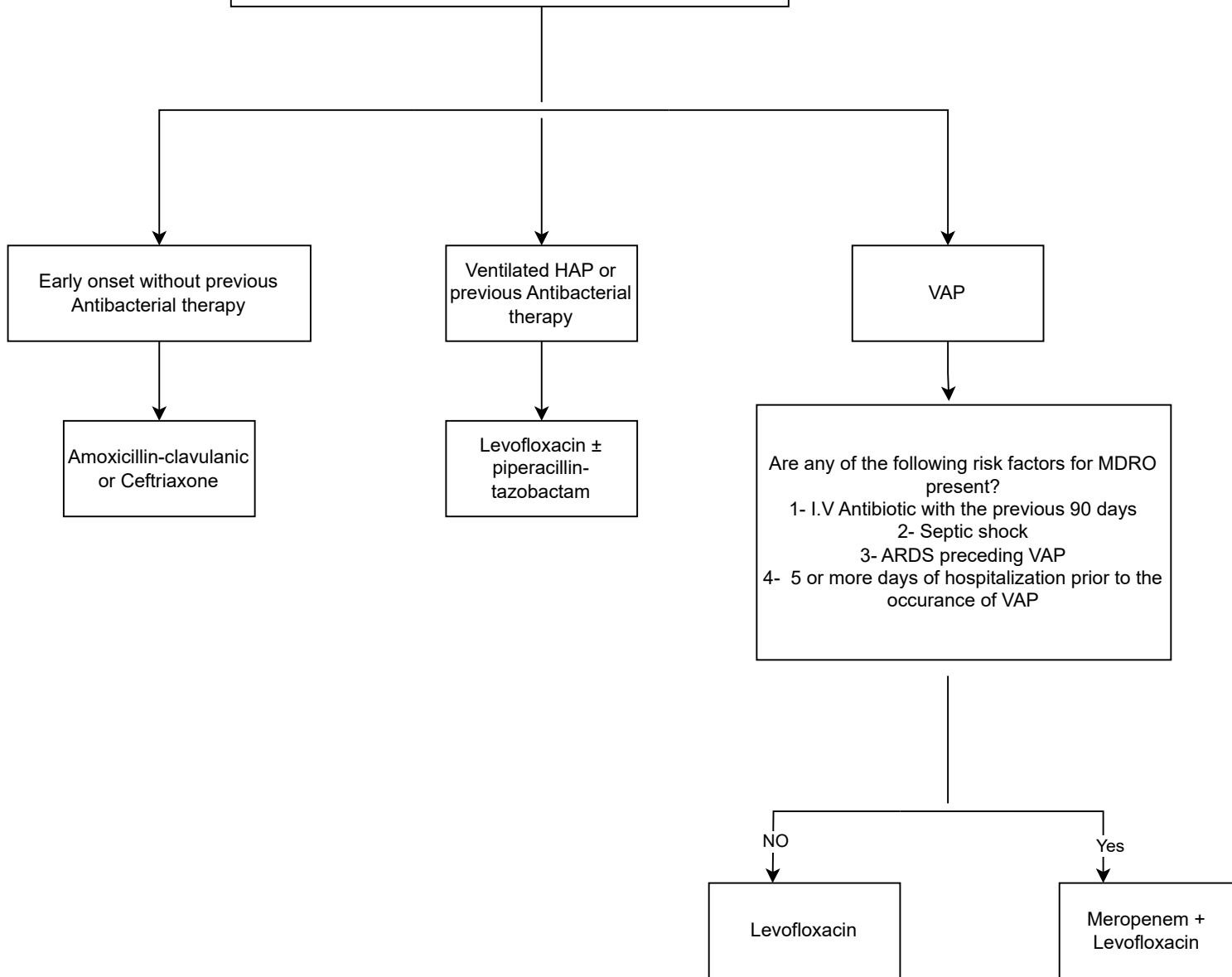

Supplement: Supplementary file 4 — Supplementary Material 4 [file 12879_2026_13358_MOESM4_ESM.pdf]

# Multidrug Resistant Organism

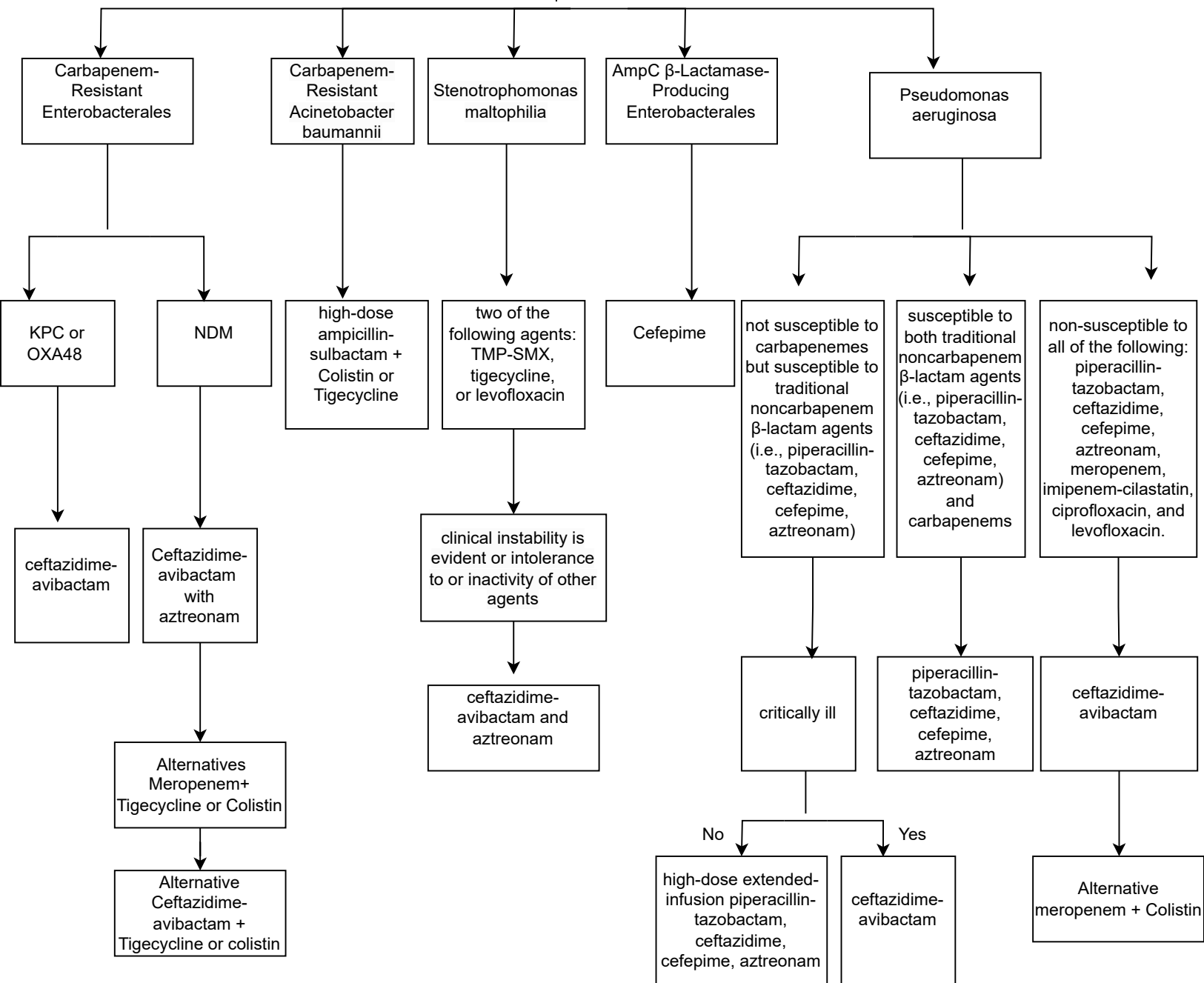

Supplement: Supplementary file 5 — Supplementary Material 5 [file 12879_2026_13358_MOESM5_ESM.pdf]

**Suspected surgical site infection**

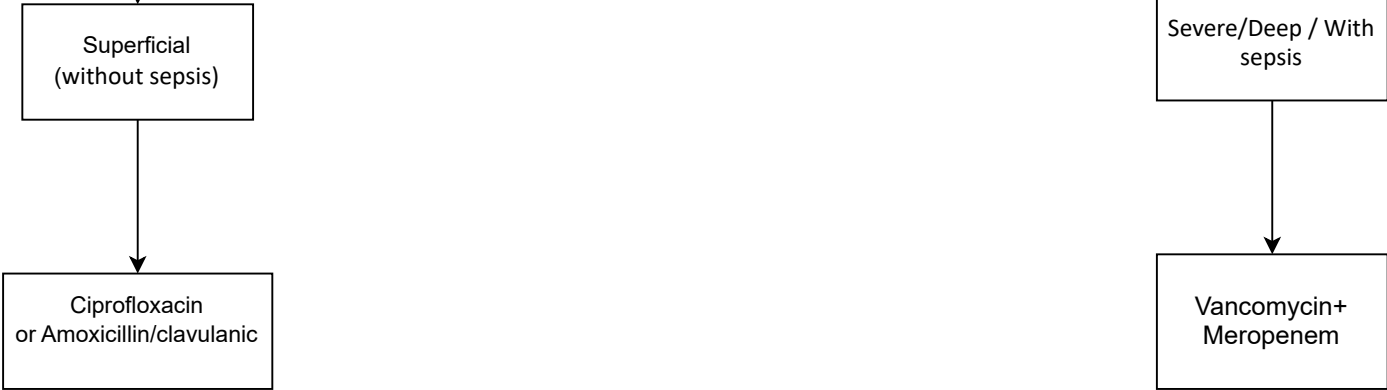

Supplement: Supplementary file 6 — Supplementary Material 6 [file 12879_2026_13358_MOESM6_ESM.pdf]
