# Supplementary material for: Evaluating the effects of antimicrobial stewardship program on antimicrobial consumption and resistance patterns: a quasi-experimental study
Source: BMC Infect Dis. 2026 May 20;26:988. doi: 10.1186/s12879-026-13358-8 (PMC13195955; doi:10.1186/s12879-026-13358-8)
Supplement: Supplementary file 7 — Supplementary Material 7 [file 12879_2026_13358_MOESM7_ESM.docx]

**Supplementary Table S1. Detailed Antimicrobial Susceptibility of Key Bacterial Isolates at Aswan Heart Center during pre-intervention (Jul– Dec 2023) and post-intervention (Jan–Jun 2024) periods of the antimicrobial stewardship program implementation.**

1. Gram-negative Bacteria

| Microbial species | No. of isolates (Pre) | No. of isolates (Post) | Antimicrobial | Susceptibility Pre-intervention n (%) | Susceptibility Post-intervention n (%) | p-value |
| --- | --- | --- | --- | --- | --- | --- |
| *Citrobacter spp.* | **2** | **2** | **Amikacin** | 2/2 (100) | 2/2 (100) |  |
|  |  |  | **Cefepime** | 0/2 (0) | 2/2 (100) | **0.33** |
|  |  |  | **Ceftazidime** | 0/2 (0) | 1/2 (50) | **1** |
|  |  |  | **Ciprofloxacin** | 0/2 (0) | 2/2 (100) | **0.33** |
|  |  |  | **Gentamicin** | 2/2 (100) | 2/2 (100) |  |
|  |  |  | **Levofloxacin** | 0/2 (0) | 2/2 (100) | **0.33** |
|  |  |  | **Imipenem** | 2/2 (100) | 2/2 (100) |  |
|  |  |  | **Meropenem** | 2/2 (100) | 2/2 (100) |  |
|  |  |  | **Piperacillin/ Tazobactam** | 2/2 (100) | 1/2 (50) | **1** |
|  |  |  | **Tigecycline** | 1/2 (50) | 2/2 (100) | **1** |
|  |  |  | **Trimethoprim/**  **Sulfamethoxazole** | 0/2 (0) | 2/2 (100) | **0.33** |
| *Escherichia coli* | **11** | **14** | **Amikacin** | 9/11 (82) | 13/14 (93) | **0.56** |
|  |  |  | **Cefepime** | 4/11 (36) | 3/14(21) | **0.65** |
|  |  |  | **Ceftazidime** | 1/11 (9) | 4/14(29) | **0.34** |
|  |  |  | **Ciprofloxacin** | 3/11 (27) | 9/14 (64) | **0.1** |
|  |  |  | **Gentamicin** | 4/11 (36) | 12/14 (86) | **0.01*** |
|  |  |  | **Levofloxacin** | 3/11 (27) | 9/14 (64) | **0.1** |
|  |  |  | **Meropenem** | 7/11 (64) | 14/14 (100) | **0.02*** |
|  |  |  | **Piperacillin/ Tazobactam** | 5/11 (46) | 10/14 (71) | **0.2** |
|  |  |  | **Tigecycline** | 11/11 (100) | 14/14 (100) |  |
|  |  |  | **Trimethoprim/Sulfamethoxazole** | 2/11 (18) | 4/14 (29) | **0.66** |

**Table 4 (continued)**

1. Gram-negative Bacteria

| Microbial species | No. of isolates (Pre) | No. of isolates (Post) | Antimicrobial | Susceptibility Pre-intervention n (%) | Susceptibility Post-intervention n (%) | p-value |
| --- | --- | --- | --- | --- | --- | --- |
| *Enterobacter spp.* | **4** | **1** | **Amikacin** | 4/4 (100) | 1/1 (100) |  |
|  |  |  | **Cefepime** | 4/4 (100) | 1/1 (100) |  |
|  |  |  | **Ceftazidime** | 4/4 (100) | 1/1 (100) |  |
|  |  |  | **Ciprofloxacin** | 4/4 (100) | 1/1 (100) |  |
|  |  |  | **Gentamicin** | 3/4 (75) | 1/1 (100) | **1** |
|  |  |  | **Levofloxacin** | 4/4 (100) | 1/1 (100) |  |
|  |  |  | **Imipenem** | 4/4 (100) | 1/1 (100) |  |
|  |  |  | **Meropenem** | 4/4 (100) | 1/1 (100) |  |
|  |  |  | **Piperacillin/ Tazobactam** | 4/4 (100) | 1/1 (100) |  |
|  |  |  | **Tigecycline** | 4/4 (100) | 1/1 (100) |  |
|  |  |  | **Trimethoprim/**    **Sulfamethoxazole** | 3/4 (75) | 1/1 (100) | **1** |
| *Klebsiella pneumoniae* | **42** | **19** | **Amikacin** | 25/42 (59) | 11/19 (58) | **1** |
|  |  |  | **Cefepime** | 7/42 (17) | 7/19(37) | **0.106** |
|  |  |  | **Ceftazidime** | 5/42 (12) | 6/19 (32) | **0.081** |
|  |  |  | **Ciprofloxacin** | 19/42 (45) | 7/19 (39) | **0.58** |
|  |  |  | **Gentamicin** | 20/42 (48) | 8/19 (42) | **0.78** |
|  |  |  | **Levofloxacin** | 19/42 (46) | 11/19 (59) | **0.41** |
|  |  |  | **Imipenem** | 17/42 (41) | 8/19 (41) | **0.78** |
|  |  |  | **Meropenem** | 21/42 (50) | 7/19 (37) | **0.41** |
|  |  |  | **Piperacillin/ Tazobactam** | 15/42 (36) | 9/19 (47) | **0.41** |
|  |  |  | **Tigecycline** | 41/42 (97) | 19/19 (100) | **1** |
|  |  |  | **Trimethoprim/**  **Sulfamethoxazole** | 17/42 (41) | 7/19 (37) | **1** |

**Table 4 (continued)**

1. Gram-negative Bacteria

| Microbial species | No. of isolates (Pre) | No. of isolates (Post) | Antimicrobial | Susceptibility Pre-intervention n (%) | Susceptibility Post-intervention n (%) | p- value |
| --- | --- | --- | --- | --- | --- | --- |
| *Proteus spp.* | 3 | 2 | **Amikacin** | 2/3 (67) | 2/2 (100) | **0.36** |
|  |  |  | **Cefepime** | 1/3 (33) | 1/2(50) | 0.7 |
|  |  |  | **Ceftazidime** | 1/3 (33) | 0/2 (0) | **0.36** |
|  |  |  | **Ciprofloxacin** | 2/3 (67) | 2/2 (100) | **0.36** |
|  |  |  | **Gentamicin** | 1/3 (33) | 2/2 (100) | **0.136** |
|  |  |  | **Levofloxacin** | 2/3 (67) | 2/2 (100) | **0.36** |
|  |  |  | **Meropenem** | 2/3 (67) | 2/2 (100) | **0.36** |
|  |  |  | **Piperacillin/ Tazobactam** | 3/3 (100) | 2/2 (100) |  |
|  |  |  | **Trimethoprim/**    **Sulfamethoxazole** | 1/3 (33) | 0/2 (0) | **0.36** |
| *Serratia marcescens* | **4** | **1** | **Amikacin** | 4/4 (100) | 1/1 (100) |  |
|  |  |  | **Cefepime** | 3/4 (75) | 1/1 (100) | **1** |
|  |  |  | **Ceftazidime** | 2/4 (50) | 1/1 (100) | **1** |
|  |  |  | **Ciprofloxacin** | 4/4 (100) | 1/1 (100) |  |
|  |  |  | **Gentamicin** | 4/4 (100) | 1/1 (100) |  |
|  |  |  | **Levofloxacin** | 3/4 (75) | 1/1 (100) | **1** |
|  |  |  | **Imipenem** | 2/4 (50) | 1/1 (100) | **1** |
|  |  |  | **Meropenem** | 4/4 (100) | 1/1 (100) |  |
|  |  |  | **Piperacillin/ Tazobactam** | 2/4 (50) | 1/1 (100) | **1** |
|  |  |  | **Tigecycline** | 2/4 (50) | 1/1 (100) | **1** |
|  |  |  | **Trimethoprim/**    **Sulfamethoxazole** | 3/4 (75) | 1/1 (100) | **1** |

**Table 4 (continued)**

B. Gram-positive Bacteria

| Microbial species | No. of isolates (Pre) | No. of isolates (Post) | Antimicrobial | Susceptibility Pre-intervention n (%) | Susceptibility Post-intervention n (%) | p- value |
| --- | --- | --- | --- | --- | --- | --- |
| *Staphylococcus aureus* | 13 | 11 | **Ampicillin** | 2/13 (15) | 2/11 (18) | 1 |
|  |  |  | **Ciprofloxacin** | 8/13 (62) | 9/11(81) | 0.38 |
|  |  |  | **Clindamycin** | 10/13 (75) | 11/11(100) | 0.2 |
|  |  |  | **Gentamicin** | 5/13 (39) | 7/11 (64) | 0.41 |
|  |  |  | **Levofloxacin** | 9/13 (69) | 9/11 (82) | 0.64 |
|  |  |  | **Moxifloxacin** | 6/13 (50) | 11/11 (100) | 0.01* |
|  |  |  | **Rifampicin** | 12/13 (92) | 11/11 (100) | 1 |
|  |  |  | **Trimethoprim/**  **Sulfamethoxazole** | 9/13 (69) | 10/11 (91) | 0.32 |

**Table 4 (continued)**

C. Fungal Isolates

| Microbial species | No. of isolates (Pre) | No. of isolates (Post) | Antimicrobial | Susceptibility Pre-intervention n (%) | Susceptibility Post-intervention n (%) | p- value |
| --- | --- | --- | --- | --- | --- | --- |
| *Candida spp.* | **9** | **4** | **Voriconazole** | 9/9 (100) | 4/4 (100) |  |
|  |  |  | **Amphotericin B** | 5/9 (56) | 4/4 (100) | **0.22** |
|  |  |  | **Fluconazole** | 5/9 (56) | 4/4 (100) | **0.22** |
|  |  |  | **Caspofungin** | 7/9 (80) | 4/4 (100) | **1** |
| *Cryptococcus spp.* | **3** | **2** | **Voriconazole** |  | 2/2 (100) |  |
|  |  |  | **Amphotericin B** | 3/3 (100) | 2/2 (100) |  |
|  |  |  | **Fluconazole** |  | 2/2 (100) |  |

Data are presented as number of susceptible isolates over total tested isolates with corresponding

percentages. P-values were calculated using Fisher’s exact test.

* Statistically significant difference (p < 0.05)
